# Comparative Mitochondrial Genome Analysis of Two Ectomycorrhizal Fungi (*Rhizopogon*) Reveals Dynamic Changes of Intron and Phylogenetic Relationships of the Subphylum Agaricomycotina

**DOI:** 10.3390/ijms20205167

**Published:** 2019-10-18

**Authors:** Qiang Li, Yuanhang Ren, Xiaodong Shi, Lianxin Peng, Jianglin Zhao, Yu Song, Gang Zhao

**Affiliations:** Key Laboratory of Coarse Cereal Processing, Ministry of Agriculture and Rural Affairs, College of Pharmacy and Biological Engineering, Chengdu University, Chengdu 610106, Sichuan, China; leeq110@126.com (Q.L.); renyuanhang@cdu.edu.cn (Y.R.); shixiaodong@cdu.edu.cn (X.S.); penglianxin@cdu.edu.cn (L.P.); jlzhao@cdu.edu.cn (J.Z.); songyu@cdu.edu.cn (Y.S.)

**Keywords:** *Rhizopogon*, mitochondrial genome, intron, gene rearrangement, evolution, phylogenetic analysis

## Abstract

In the present study, we assembled and compared two mitogenomes from the *Rhizopogon* genus. The two mitogenomes of *R. salebrosus* and *R. vinicolor* comprised circular DNA molecules, with the sizes of 66,704 bp and 77,109 bp, respectively. Comparative mitogenome analysis indicated that the length and base composition of protein coding genes (PCGs), rRNA genes and tRNA genes varied between the two species. Large fragments aligned between the mitochondrial and nuclear genomes of both *R. salebrosus* (43.41 kb) and *R. vinicolor* (12.83 kb) indicated that genetic transfer between mitochondrial and nuclear genomes has occurred over evolutionary time of *Rhizopogon* species. Intronic regions were found to be the main factors contributing to mitogenome expansion in *R. vinicolor*. Variations in the number and type of introns in the two mitogenomes indicated that frequent intron loss/gain events occurred during the evolution of *Rhizopogon* species. Phylogenetic analyses based on Bayesian inference (BI) and Maximum likelihood (ML) methods using a combined mitochondrial gene set yielded identical and well-supported tree topologies, wherein *Rhizopogon* species showed close relationships with Agaricales species. This is the first study of mitogenomes within the genus *Rhizopogon*, and it provides a basis for understanding the evolution and differentiation of mitogenomes from the ectomycorrhizal fungal genus.

## 1. Introduction

The genus *Rhizopogon*, (Boletales, Basidiomycota), is an important ectomycorrhizal fungal group, which was widely distributed in Europe and North America [1,2,3,4,5]. The genus forms hypogeous sporocarps, commonly referred to as ′false truffles′. *Rhizopogon* species form ectomycorrhizal associations with trees in the family *Pinaceae*, primarily *Pinus* and *Pseudotsuga* spp. Through their ectomycorrhizal relationships, *Rhizopogon* species obtains carbon sources necessary for growth, and in exchange they provide trees with access to water and mineral nutrients [1]. In addition, ectomycorrhizal fungi, e.g., *Rhizopogon* spp., promote plant growth, enhance plant resistance to stress and maintain the forest ecosystem [6,7,8]. The genus *Rhizopogon* is diverse, with more than 150 species described [3,9,10]. However, it is difficult to identify and classify *Rhizopogon* species accurately because of the limited morphological characteristics that can be distinguished and the overlapping of some morphological features. The uniparental inheritance, rapid evolution rate and availability of molecular markers [11] have made mitochondrial genome powerful tools for studying evolutionary biology, phylogenetics and taxonomy of Eukaryotes [12,13]. Up to now, no mitochondrial genomes have been assembled from sequence data in the genus, which limits our understanding of this important ectomycorrhizal fungal group.

Mitochondria are the main chemical energy suppliers for aerobic, free-living eukaryotes, and play an important role in growth, development, aging and stress resistance [14,15]. Mitochondria contain their own genomes, commonly known as the "second genome" of eukaryotes [16], and hereafter referred to as mitogenomes. There are 20–36 tRNA genes, 1–2 rRNA genes, a series of genes for energy metabolism, and several regulatory genes found in eukaryotic mitogenomes [17]. Mitogenomes from different eukaryotic groups have undergone tremendous diversification in genome size, and in gene content, structure, and arrangement [18,19,20]. Mitogenomes of animals have been extensively studied, and more than 8000 animal mitogenomes have been reported in the NCBI database (https://www.ncbi.nlm.nih.gov/genome/browse#!/organelles/), and these have promoted the development of animal taxonomy and genetics [21,22]. However, mitogenomes in fungi, especially in Agaricomycotina, have been less studied than the animal counterpart [23], although they were initially targeted in early work using mt-rRNA genes or selected mt-protein genes. As the largest mushroom-forming fungal group, less than 80 Agaricomycotina mitogenomes have been reported, which limits the understanding of the “second genome” of the mushroom-forming fungi. It was reported that the evolution rate of mitogenomes from fungi was intermediate to that of animals (highest mutation rate) and plants (lowest mutation rate) [24]. Previous studies found that mitogenomes of fungi differed greatly in genome size, content of repeat sequence, gene arrangement, and genome organization [25,26,27]. However, most Agaricomycotina mitogenomes were found to contain 14 core protein coding genes (PCGs), including *atp6, atp8, atp9, cob, cox1, cox2, cox3, nad1, nad2, nad3, nad4, nad4L, nad5*, and *nad6*, for energy metabolism plus one *rps3* gene for transcriptional regulation; collectively we call this set of the genes the core PCGs of Agaricomycotina. The features and variations of mitogenome size, organization and gene content of *Rhizopogon* species are helpful to reveal the evolution and phylogeny of *Rhizopogon* species.

Introns are common in mitogenomes of fungi, and they are considered to be one of the main factors contributing to variations in the size and organization of fungal mitogenomes [28,29]. Introns in the mitogenomes of fungi can be divided into two groups, namely, I and II. The *cox1* gene was found to be the main host gene of introns in fungal mitogenome [30]. In addition, introns were also detected in other mitochondrial genes, such as the *cox2*, *cob*, *nad1*, *nad5* and *rnl* [31]. Mitochondrial introns can behave as mobile genetic elements [32,33]. Homologous introns often have the same insertion sites in the coding region of host genes and also have high sequence similarities [30]. Introns can be divided into different position classes (Pcls) according to their insertion sites in the coding region of PCGs, while the same Pcls generally contain homologous intronic ORFs [30], which encode homing endonucleases. The number and type of introns varied greatly between different fungal species [30]. Up to known, the intron information in *Rhizopogon* genus has not been revealed.

In the present study, the complete mitogenomes of *R. salebrosus* and *R. vinicolor* were assembled and annotated. The gene content, tRNA structure, repeat sequences, intron information and genome organization of the two mitogenomes were analyzed. We also compared the two mitogenomes to identify variations and similarities in genome organization, gene content, and gene order. In addition, the phylogenetic relationships among various Agaricomycotina species were analyzed based on combined mitochondrial gene sets. As an important ectomycorrhizal fungus, the *Rhizopogon* species plays an important role in maintaining the forest ecosystem and promoting a natural carbon and nitrogen cycle. The mitogenomes of the two *Rhizopogon* species in this study further our understanding of the genetics, taxonomy, and evolutionary biology of the ectomycorrhizal fungi, and other related fungal species.

## 2. Results

### 2.1. Features and Protein Coding Genes of the Rhizopogon Mitogenomes

**Figure 1:**
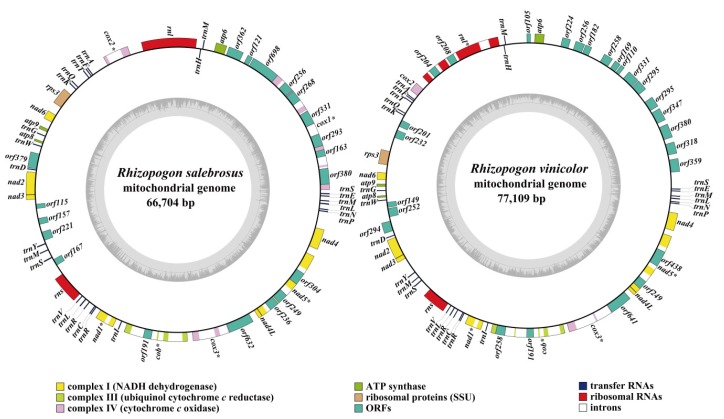
Circular maps of the mitogenomes of two *Rhizopogon* species. Genes are represented by different colored blocks. Colored blocks outside each ring indicate that the genes are on the direct strand, while colored blocks within the ring indicates that the genes are located on the reverse strand. The circle inside the GC content graph marks the 50% threshold.

The complete mitogenomes of *R. salebrosus* and *R. vinicolor* were composed of circular DNA molecules, with the total size of 66,704 bp and77,109 bp, respectively (Figure 1). The GC content of *R. salebrosus* and *R. vinicolor* mitogenomes were 21.45% and 21.46%, respectively. Both mitogenomes contained negative AT skews and positive GC skews (Table S1). A total of 24 and 22 non-intronic ORFs were detected in the mitogenomes of *R. salebrosus* and *R. vinicolor*, respectively, including 15 core PCGs (*atp6*, *atp8*, *atp9*, *cob*, *cox1*, *cox2*, *cox3*, *nad1*, *nad2*, *nad3*, *nad4*, *nad4L*, *nad5*, *nad6*, and *rps3*) and several non-core PCGs (Table S2). The *R. salebrosus* mitogenome contains 9 non-intronic PCGs, 3 of which encoded B-type DNA polymerase, and the other six have unknown functions. The *R. vinicolor* mitogenome contained 7 additional non-intronic ORFs, which included 2 B-type DNA polymerase genes and 5 PCGs with unknown function. We detected 16 and 24 introns in the mitogenomes of *R. salebrosus* and *R. vinicolor*, respectively. All of these introns belonged to the group I. In addition, 10 and 19 intronic ORFs were detected in the mitogenome of *R. salebrosus* and *R. vinicolor*, respectively, which encoded LAGLIDADG or GIY-YIG homing endonucleases.

### 2.2. RNA Genes in the Rhizopogon Mitogenomes

**Figure 2:**
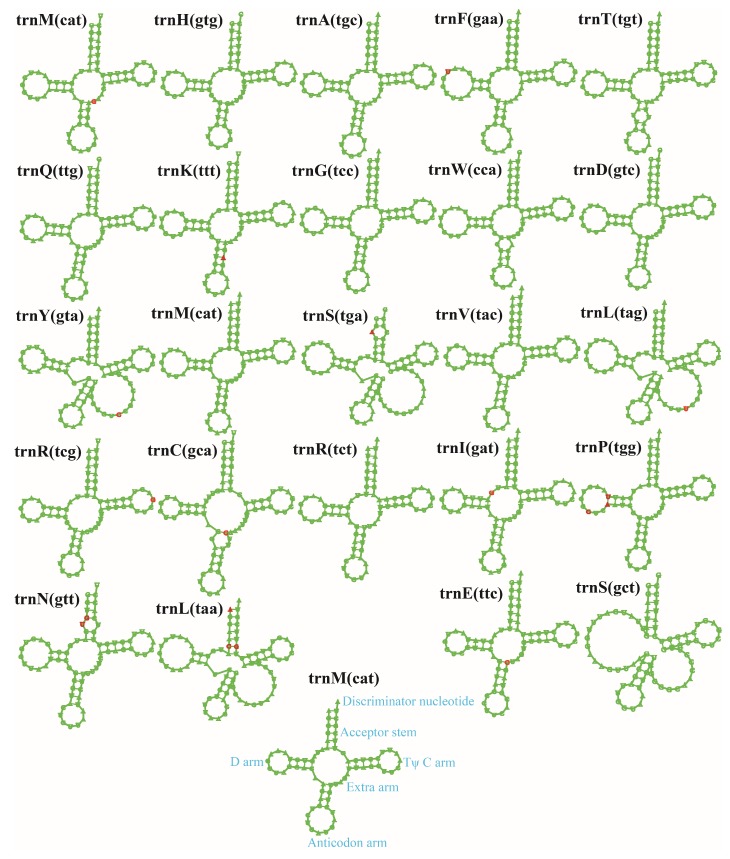
Putative secondary structures of the 25 tRNA genes identified in the mitogenomes of two *Rhizopogon* species. Residues conserved across the two mitogenomes are shown in green, while variable sites are shown in red. All genes are shown in order of occurrence in the mitogenome of *R. salebrosus*, starting from *trnM*.

Two rRNA genes were detected in the mitogenomes of *R. salebrosus* and *R. vinicolor*, namely the small subunit ribosomal RNA (*rns*), and the large subunit ribosomal RNA (*rnl*) (Table S2). Twenty-five tRNA genes were detected in the two mitogenomes, which were folded into classical cloverleaf structures (Figure 2). The two mitogenomes both contained 2 isoacceptors with different anticodons for serine, arginine, and leucine, and 3 isodecoders with the same anticodon for methionine. The length of individual tRNAs ranged from 71 bp to 88 bp, which was mainly due to variations of extra arms. Of the 25 tRNA genes detected in the two mitogenomes, 13 contained sites that varied between the two mitogenomes. The most common variable site was located on the acceptor stem (six sites varied between the two mitogenomes), followed by the D arm that contained four variable sites.

### 2.3. Overlapping Nucleotides and Composition of Mitogenomes

We detected four overlapping nucleotides in the mitogenome of *R. salebrosus*, with the largest overlapping nucleotides located across the neighboring genes *cox3* and orf632 (−85 bp) (Table S2). Three sets of overlapping nucleotides were detected in the mitogenome of *R. vinicolor*, and the largest set of overlapping nucleotides was located between *cox3* and orf641 (−85 bp). A total of 18,577 bp and 18,040 bp of intergenic sequences were identified in the mitogenome of *R. salebrosus* and *R. vinicolor*, respectively. The length of intergenic sequences ranged from 37 bp to 1791 bp, and the longest intergenic sequence was located between orf105 and *trnM* in the *R. vinicolor* mitogenome.

**Figure 3:**
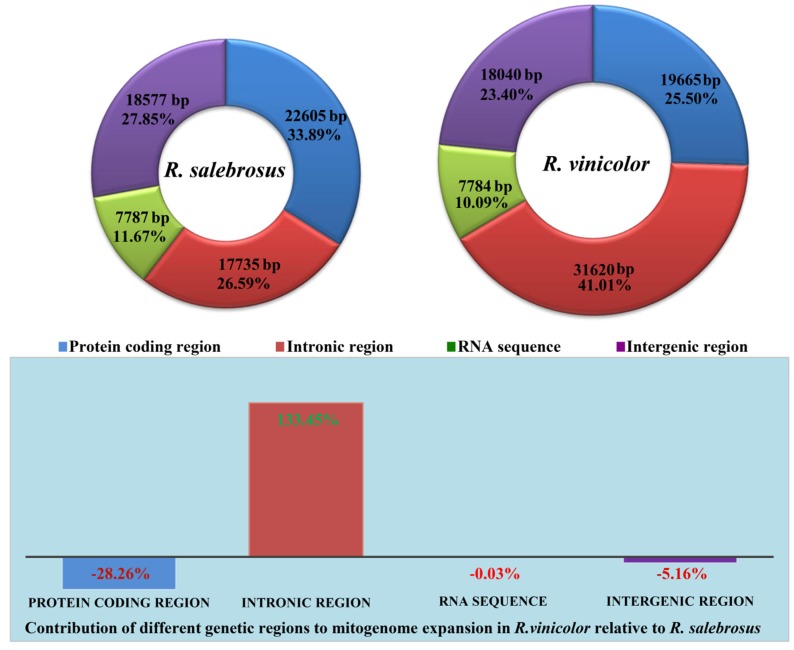
The protein-coding, intronic, intergenic, and RNA gene region proportions of the entire mitogenomes of the two *Rhizopogon* species. The bottom panel shows the contribution of different gene regions to the expansion of the *R. vinicolor* mitogenome.

The protein coding region accounted for the largest proportion of the *R. salebrosus* mitogenome, totaling 33.89% (Figure 3). Intronic regions occupied the largest proportion of the *R. vinicolor* mitogenome, accounting for 41.01%. RNA coding regions (tRNA and rRNA) accounted for the smallest proportion of the two mitogenomes (10.09–11.67%). The mitogenome of *R. vinicolor* was 10,405 bp larger than that of *R. salebrosus*. Intronic regions contributed the most to the mitogenome expansion in *R. vinicolor*, totaling 133.45% of the size difference (Figure 3). Protein coding regions contributed −28.26% of the increased size, and intergenic regions contributed −5.16% of the *R. vinicolor* mitogenome expansion. These results indicated that the expansion of the *R. vinicolor* mitogenome was primarily due to the increase of intronic regions.

### 2.4. Codon Usage Analysis

All the 12 Agaricales and 2 Boletales species tested used the universal mitochondrial code. As shown in Table S3, most core PCGs used ATG as start codons and TAA as stop codons in the two *Rhizopogon* species. However, *cob* genes in the two *Rhizopogon* species used TAG as stop codons, just like the *cob* gene of *Agaricus bisporus* [30]. *Cox1* genes of the two *Rhizopogon* species used TTG as start codons, just as *Moniliophthora* species [34,35] from the order Agaricales.

**Figure 4:**
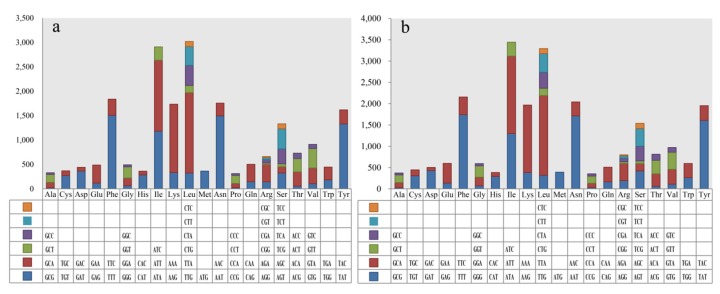
Codon usage in the mitogenomes of two *Rhizopogon* species. Frequency of codon usage is plotted on the y-axis. (**a**) *R. salebrosus*; (**b**) *R. vinicolor*.

Codon usage analysis indicated that the most frequently used codons in the two mitogenomes were TTA (for leucine; Leu), TTT (for phenylalanine; Phe), AAT (for asparagine; Asn), ATT (for isoleucine; Ile), AAA (for lysine; Lys), and TAT (for tyrosine; Tyr) (Figure 4). The frequent use of A and T in codons contributed to the high AT content in the *Rhizopogon* mitogenomes (average: 78.55%).

### 2.5. Repetitive Sequences Analysis

Comparing the whole mitogenomes of *R. salebrosus* and *R. vinicolor* with themselves via BLASTn searches, we identified 12 repeat sequences in the mitogenome of *R. vinicolor* (Table S4). No repeat sequence was identified in the *R. salebrosus* mitogenome. The length of repeat sequences in the *R. vinicolor* mitogenome ranged from 39 bp to 810 bp, with pair-wise nucleotide similarities ranging from 80.37% to 100%. The largest repeats were observed in the intergenic region between orf105 and *trnM*, as well as in the intergenic region between orf232 and *rps3*. The second largest repeats were found located in the intergenic region between orf105 and *trnM*, as well as in intergenic region between *trnK* and orf201, with each repeating sequence 163 bp long. Repeat sequence accounts for 3.01% of the *R. vinicolor* mitogenome.

A total of 63 and 64 tandem repeats were identified in the mitogenomes of *R. salebrosus* and *R. vinicolor*, respectively (Table S5). The longest tandem repeat sequence was observed in the *R. vinicolor* mitogenome, comprising 116 bp, which was located in the intergenic region between orf249 and orf438. Most of the tandem repeats were duplicated once or twice in the two mitogenomes, with the highest replication number (13) in the *R. vinicolor* mitogenome. Tandem repeat sequences accounted for 5.36% and 4.39% of the *R. salebrosus* and *R. vinicolor* mitogenomes, respectively. REPuter identified 6 forward, and 1 reverse repeats in the mitogenome of *R. salebrosus*, accounting for 0.73% of the entire mitogenome (Table S6). A total of 34 forward, 4 palindromic, and 12 reverse repeats were detected in the mitogenome *R. vinicolor*, which accounted for 2.53% of the total mitogenome.

To identify gene segments that may have been transferred between the nuclear and mitogenomes, we blasted the two mitogenomes against their nuclear genomes. A total of 55 and 30 aligned fragments were detected in the mitogenome of *R. salebrosus* and *R. vinicolor*, respectively (Table S7). The length of these aligned fragments ranged from 30 bp to 3005 bp, with sequence identities between 80.37% and 100%. The largest aligned fragment was found located in the protein coding regions of *cox1* gene in the *R. salebrosus* mitogenome. The largest aligned fragment in the *R. vinicolor* mitogenome (2376 bp) was found encompassed in the protein coding regions orf232 and *rps3*, as well as the intergenic region between them. The presence of large fragments that aligned between the mitochondrial and nuclear genomes of the two *Rhizopogon* mitogenomes indicated that genetic transfer between mitochondrial and nuclear genomes may have occurred over evolutionary time of *Rhizopogon* species.

### 2.6. Variation, Genetic Distance, and Evolutionary Rates of Core Genes

**Figure 5:**
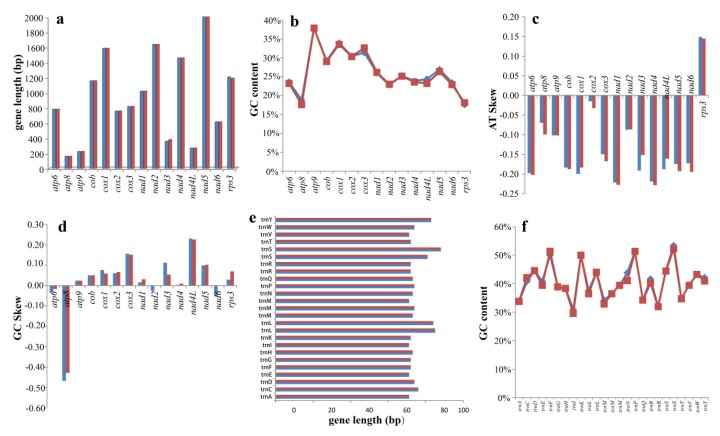
Variation in the length and base composition of each of 15 protein-coding genes (PCGs) and 25 tRNA genes between two *Rhizopogon* mitogenomes. *R. salebrosus* is represented in blue and *R. vinicolor* is represented in red. (**a**) PCG length variation; (**b**) GC content of the PCGs; (**c**) AT skew; (**d**) GC skew; (**e**) lengths of shared tRNA genes; (**f**) GC content of shared tRNA genes.

Among the 15 core PCGs detected, 3 PCGs varied in length between the two *Rhizopogon* species, including *nad3*, *nad4* and *rps3* (Figure 5). The length of *nad3* gene had the greatest variation, and there were 7 amino acid variations between the two *Rhizopogon* species. The GC content for each of the core PCGs varied between the two *Rhizopogon* species, except for that of the *atp9* gene. AT skews of core PCGs used for energy metabolism were negative in both *Rhizopogon* species, while *rps3*, which is used for transcriptional regulation, was positive in the two mitogenomes. The GC skews of core PCGs in the two mitogenomes were variable. The *atp6* and *atp8* genes contained negative GC skews in both species. GC skews in *nad2* and *nad6* genes of the *R. salebrosus* mitogenome were negative, while they were 0 in the two genes of the *R. vinicolor* mitogenome. The length of tRNA genes was conservated between the two *Rhizopogon* species. The *trnS* gene had the longest length among the 25 tRNA genes detected in the two mitogenomes, followed by the *trnL* gene, due primarily to their relatively long extra arm. Ten of the 25 tRNA genes each showed variations in GC content between the two *Rhizopogon* species, suggesting that PCGs and tRNA genes in *Rhizopogon* species undergo frequent base variation.

**Figure 6:**
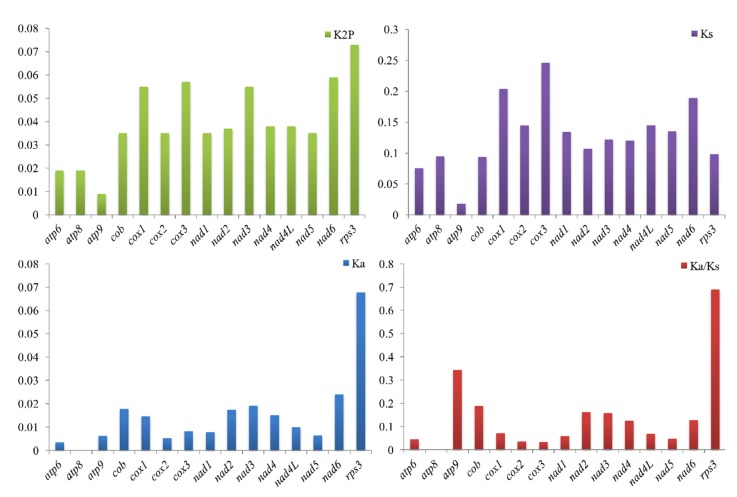
Genetic analysis of 15 protein coding genes conserved in two *Rhizopogon* mitogenomes. K2P — the Kimura−2-parameter distance; Ka — the mean number of nonsynonymous substitutions per nonsynonymous site; Ks — the mean number of synonymous substitutions per synonymous site.

Among the 15 core PCGs detected, *rps3* gene had the largest K2P genetic distance between the two *Rhizopogon* species, followed by *nad6* gene (Figure 6). The K2P genetic distance of *atp9* gene between two *Rhizopogon* species was the smallest, indicating that it was highly conserved between *Rhizopogon* species. The *cox3* gene had the largest synonymous substitutions rate (Ks) among the 15 core PCGs, while *atp9* had the smallest Ks value. The nonsynonymous substitution rate (Ka) of *atp8* gene was 0, while that of *rps3* gene was the largest in the *Rhizopogon* species. The Ka/Ks values of the 15 core PCGs ranged from 0 to 0.69, indicating that these genes were subjected to purifying selection.

### 2.7. Dynamic Changes of Introns in Rhizopogon Mitogenomes

A total of 40 introns were detected in mitogenomes of the two *Rhizopogon* species, which were distributed in *cox1*, *cox2*, *cox3*, *cob*, *nad1*, *nad5* and *rnl* genes. The *cox1* gene hosted the largest number of introns in *Rhizopogon* species with 18 of 40 introns located in it (Table S8). The *cob* gene hosted the second largest number with 7 introns. Each intron harboured 0–2 homing endonuclease genes, including the LAGLIDADG type 1 homing endonuclease, the LAGLIDADG type 2 homing endonuclease and GIY-YIG endonuclease. Several intron types were detected in the mitogenomes of *Rhizopogon* species, with the most common type being Group IB, followed by the Group ID. Two introns with novel types were found in *cob* and *rnl* genes of the two *Rhizopogon* mitogenomes. The 18 introns in *cox1* genes of the two *Rhizopogon* species could be classified into 14 Pcls according to their insertion positions in the coding region of PCGs. Among them, 11 were homologous with Pcls from previously reported mitogenomes [30], while the other three were unknown Pcls that were unique in one of the two *Rhizopogon* species. Pcls K, S, AC and AD were detected in both *Rhizopogon* species and were widely distributed in other species of fungi. Pcl P was detected only in the *cox1* gene of *R. salebrosus*, but were also present in distantly related species, such as *Agaricus bisporus* [30] and *Pleurotus ostreatus* [25]. Pcls D, G, H, N, AA, AI found in the mitogenome of *R. vinicolor*, were also present in plants such as *Marchantia polymorpha*, *Beta vulgaris*, *Plantago atrata*, and others [30]. In *cox2* gene, *R. salebrosus* contained an intron inserted at 543 nt of the protein coding region, and homologous intron was not detected in another *Rhizopogon* species *R. vinicolor*. Non-homologous introns between the two mitogenomes accounted for 50%, 14.29%, 60% and 100% of the total introns in *cox3*, *cob*, *nad5* and *rnl* genes, respectively. Variations of the number and Pcl of introns between the two mitogenomes indicated that frequent intron loss/gain occurred in the evolution of *Rhizopogon* species. Homologous introns in *Rhizopogon* species were also found in distant species from other orders, indicating possible horizontal gene transfer events.

### 2.8. Gene Arrangement and Phylogenetic Analysis

The arrangement of mitochondrial genes could be used as a reference for studying the phylogenetic status and evolution of species. In the present study, we used Mauve to analyze the collinearity of mitogenomes between two *Rhizopogon* species and 16 other species from Agaricomycetes. The results showed that the arrangement of mitochondrial genes in Agaricomycetes species were highly variable (Figure S1). Several homologous regions were detected between the 18 species of Agaricomycetes. The size and location of these homologous regions varied greatly between the 18 species, even between species from the same genera, such as the *Cantharellus*, *Pleurotus* and *Ganoderma* genera. However, a high degree of collinearity was found between two species from the genus *Rhizopogon*. The results showed that *Rhizopogon* species were conservative in gene arrangement during evolution.

**Figure 7:**
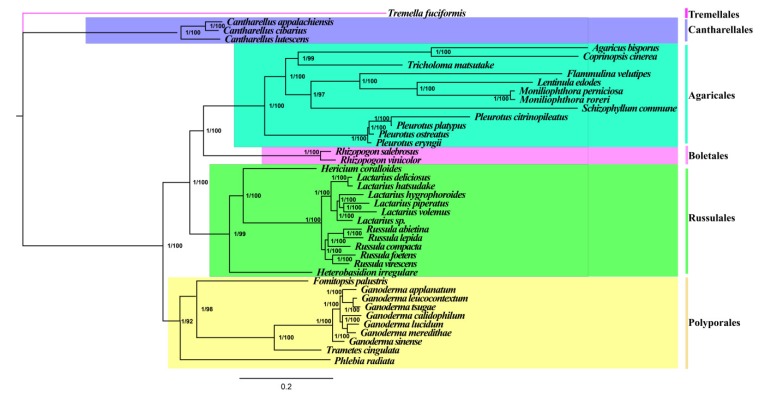
Molecular phylogeny of 41 Agaricomycotina species based on Bayesian inference (BI) and Maximum likelihood (ML) analysis of 15 protein coding genes and two rRNA genes. Support values are Bayesian posterior probabilities (before slash) and bootstrap (BS) values (after slash). Species and NCBI accession numbers for genomes used in the phylogenetic analysis are provided in Supplementary Table S9.

Phylogenetic analysis based on BI and ML methods using the combined mitochondrial gene set (15 core PCGs + 2 rRNA genes) yielded identical and well-supported tree topologies (Figure 7). All major clades within the trees were well supported (BPP = 1.00; BS ≥ 97). Based on the phylogenetic tree, the 41 Agaricomycotina species could be divided into six major clades, corresponding to the orders Tremellales, Cantharellales, Agaricales, Boletales, Russulales, and Polyporales (Table S9). Cantharellales species in Agaricomycotina were distant from other Agaricomycetes species, indicating their early differentiation from other species in Agaricomycetes. Phylogenetic analysis also showed that Boletales had a relatively recent phylogenetic relationship with Agaricales and Polyporales. The analysis further indicated that *R. salebrosus* were a sister species to *R. vinicolor*. Phylogenetic analyses based on mitochondrial genes were consistent with that based on nuclear genes [36]. The results indicated that the combined mitochondrial gene dataset was suitable as a reliable molecular marker for the analysis of phylogenetic relationships between Agaricomycotina species.

## 3. Discussion

The mitogenome size of fungi varied greatly compared with that of other eukaryotic groups [37]. Interspecific and intraspecific fungal species exhibited high variabilities in mitogenome size, which was mainly due to the variation of introns, the accumulation of repetitive sequences, intergenic sequences and horizontal transferred genes [31,34,38]. In the present study, we found that mitogenomes of the two *Rhizopogon* species were of medium size, smaller than those of *Phlebia radiate* [30,39], *A. bisporus* [30] and *Lentinula edodes* [40], and larger than those of *Coprinopsis cinerea* [41] and *Schizophyllum commune* [42], which all belonged to the Agaricomycotina. Mitogenome size also varied greatly between the two species in the *Rhizopogon* genus. Mitogenome expanded 10,405 bp in *R. vinicolor* species, and intron was found to be the most important factor leading to mitogenome expansion in *R. vinicolor*. The result was consistent with previous studies that intron variation was one of the main factors contributing to the variability of fungal mitogenome size [30,43].

Since the mitogenome of eukaryotes was obtained from the common ancestor Alphaproteobacteria [19,44], it has evolved differently in multiple lineages to form the morphology and structure it now possesses. In the course of evolution, most of the mitochondrial genes were transferred to the nuclear genome [45]. Only genes, such as the mitochondrial respiratory chain related genes, tRNA genes, rRNA genes, and some regulatory genes were retained in the mitogenome. Up to now, almost all published basidiomycete mitogenomes contained 15 core PCGs (*atp6*, *atp8*, *atp9*, *cob*, *cox1*, *cox2*, *cox3*, *nad1*, *nad2*, *nad3*, *nad4*, *nad4L*, *nad5*, *nad6*, *rps3*). However, we found that the length and base composition of the core PCGs varied greatly between mitogenomes of the two *Rhizopogon* species, as well as other basidiomycetes [46,47]. In addition, different core PCGs exhibited variable genetic distances between the two *Rhizopogon* mitogenomes, which shows that the evolutionary rates of these core genes vary. It was also found that the core PCGs were subjected to purifying selection in the two *Rhizopogon* species. The evolutionary characteristics of mitochondrial core PCGs provided important information for studying phylogenetic and genetics of fungal species. In addition, some non-conservative PCGs were detected in fungal mitogenomes, including DNA polymerase, RNA polymerase and some PCGs with unknown function. Further studies are needed to elucidate the origin, distribution, function and phylogenetic relationships of these unknown functional genes to fully understand the evolution and function of mitogenome. Different numbers of tRNA genes had also been detected in fungal mitogenomes, which varied in base composition and gene length. In this study, we found that 13 of the 25 tRNA genes shared by *Rhizopogon* species had site variations between the two *Rhizopogon* species. Mitochondrial tRNA mutation has been found to affect amino acid transport efficiency and protein synthesis in other eukaryotes [48,49]. The effects of tRNA variations on fungal growth and mitochondrial function are still unknown.

The mitochondrial gene arrangement could also be used to reveal the evolutionary and phylogenetic relationships of species [50,51]. Mitochondrial gene rearrangements have been extensively studied in animals, and several models have been proposed to reveal the mechanism of mitogenome rearrangements [52,53]. However, compared with animals, the mitochondrial gene order of fungi is highly variable, even among closely related species [25]. Up to now, mitochondrial gene rearrangements have been detected in most of the mushroom-forming (Agaricomycetes) fungal mitogenomes, such as *Pleurotus* spp. [25], *Lyophyllum* spp. [31], *Lactarius* spp. [26], *Russula* spp. [27], and *Cantharellus* spp. [54]. This may be due to the accumulation of repetitive sequences in mitogenomes of these fungi [24]. However, we found a high degree of collinearity between the two *Rhizopogon* species, suggesting that the gene arrangement of the two *Rhizopogon* species was conservative in the evolutionary process. A small number of repetitive sequences in mitogenomes were considered to be one of the reasons for conservative gene arrangement in *Rhizopogon* species.

Introns are considered as mobile genetic elements in mitogenomes of eukaryotes. The dynamic changes of introns could affect the organization and size of mitogenomes [29,33]. Introns could be classified into different Pcls according to their insertion positions in the coding region of PCGs [30]. Introns belonging to the same Pcl were considered to be orthologous and had high sequence similarities. Homologous intronic ORFs were found in introns from the same Pcls. In the present study, we found that the number and Pcl of introns were highly variable between the two *Rhizopogon* species, suggesting that frequent intron loss/gain occurred during the evolution of *Rhizopogon* species. In addition, introns homologous to *Rhizopogon* species were detected in other distant species, indicating possible horizontal gene transfer events.

The genus *Rhizopogon* shows high species diversity, which are widely distributed in Europe and North America [5]. Over 150 species have been described in the *Rhizopogon* genus. Because *Rhizopogon* species are easily cultured, they have been frequently used to study the evolution and genetics of ectomycorrhizal fungi [55]. Considering that macrofungi have limited identifiable morphological characteristics, it is difficult to classify and identify species by morphology alone accurately [56]. The introduction of molecular markers, such as rDNA ITS sequence, nuc-ssu, nuc-lsu, *atp6*, mt-lsu and so on [2,3,9], has promoted the development of population genetics, taxonomy and biogeography of *Rhizopogon* species. However, as an important group of ectomycorrhizal fungi, no mitogenome has been reported in the *Rhizopogon* genus or even the Rhizopogonaceae family, which limits a comprehensive understanding of the genetics and phylogeny of this ectomycorrhizal fungal genus. In this study, we obtained an identical and well-supported tree topology based on the combined mitochondrial gene set, wherein the *Rhizopogon* genus was found having a close phylogenetic relationship with Agaricales species. The results showed that mitochondrial genes were reliable tools for studying phylogenetic relationships of Agaricomycotina. Fungi are widely distributed in the world and have complex taxonomic systems. More fungal mitogenomes are needed in further study to reveal the origin, evolution and genetic differentiation of fungi.

## 4. Materials and Methods

### 4.1. De Novo Assembly and Annotation of Mitogenomes

Raw data of the *R. salebrosus* and *R. vinicolor* genome sequencings were downloaded from the Joint Genome Institute (JGI) database. These sequence data were produced by the US Department of Energy Joint Genome Institute (https://www.jgi.doe.gov/) in collaboration with the user community [55]. Clean reads were obtained through a series of quality control steps from the raw data. De novo assembly of the two mitogenomes was performed as implemented by SPAdes 3.9 [57] using the obtained clean data. The software MITObim V1.9 [58] was used to fill gaps among contigs. The complete mitogenomes of *R. salebrosus* and *R. vinicolor* were obtained, and then annotated combining the results of the MFannot tool [59] and MITOS [60], both based on the genetic code 4. The PCGs, tRNA genes and rRNA genes were preliminarily annotated based on the two software. PCGs were then predicted or modified using the NCBI Open Reading Frame Finder [61] and annotated via BLASTP searches against the NCBI non-redundant protein sequence database [62]. PCGs with no significant similarity to previously characterized proteins were annotated as hypothetical proteins. tRNA genes were also predicted using the tRNAscan-SE 1.3.1 program [63]. Intron-exon borders of PCGs were verified using exonerate v2.2 [64].

### 4.2. Sequence Analysis

Base composition of the two mitogenomes was analyzed using the DNASTAR Lasergene v7.1 (http://www.dnastar.com/). Strand asymmetry of the two mitogenomes was assessed according to the following formulas: AT skew = [A − T] / [A + T], and GC skew = [G − C] / [G + C] [65]. Genetic distances between each pair of the 15 core PCGs (*atp6, atp8, atp9, cob, cox1, cox2, cox3, nad1, nad2, nad3, nad4, nad4L, nad5, nad6,* and *rps3*) were calculated with MEGA v6.06 [66], using the Kimura−2-parameter (K2P) model. We used the DnaSP v6 [67] to calculate the nonsynonymous substitution rate (Ka) and the synonymous substitution rate (Ks) for all the 15 core PCGs in the two *Rhizopogon* mitogenomes. Codon usage analysis was conducted using the Sequence Manipulation Suite [68], based on the genetic code 4. Genome synteny of the two *Rhizopogon* mitogenomes and representative species from other genera were analyzed using the Mauve v2.4.0 [69].

### 4.3. Repetitive Elements Analysis

To determine whether there is intra-genomic duplication of large fragments and interspersed repeats in the two *Rhizopogon* mitogenomes, BLASTn searches of the whole mitogenomes against themselves were performed at an E value of < 10^−10^. Tandem repeats (> 10 bp in length) in the two mitogenomes were detected using the Tandem Repeats Finder [70] with default parameters. In addition, repeated sequences were searched by REPuter [71] to identify forward (direct), reverse, complemented, and palindromic (reverse complemented) repeats in the two mitogenomes. To identify any gene segments that may have transferred between the mitochondrial and nuclear genomes of the two species, we performed BLASTn searches of the two mitogenomes against their previously published nuclear genomes (PVYR00000000.1 and LYZI00000000.1) [55].

### 4.4. Intron Analysis

Group I introns in the two *Rhizopogon* mitogenomes were classified into different position classes (Pcls) according to the method described by Férandon et al. [30]. Each Pcl was constituted by introns inserted at the same position in the coding region of the PCGs. Introns of the same Pcls and that contained high sequence similarities were considered to be homologous. Different Pcls showed low sequence similarities and contained non-homologous mobile genetic elements. The Pcls of *cox1* gene were named by letters according to the similarity with the described Pcls [30]. Pcls of *cox2, cox3, cob, nad1, nad5, rnl* genes were named by number according to the insert position in the coding region of host gene.

### 4.5. Phylogenetic Analysis

In order to investigate the phylogenetic status of the two *Rhizopogon* species among Agaricomycotina subphylum, we constructed a phylogenetic tree of 41 Agaricomycotina species based on the combined mitochondrial gene set (15 core PCGs + two rRNA genes). Single mitochondrial genes were first aligned using MAFFT v7.037 [72] and then these alignments were concatenated using SequenceMatrix v1.7.8 [73]. A preliminary partition homogeneity test was carried out to detect potential phylogenetic conflicts between different genes. Best-fit models of evolution and partitioning schemes for the gene set were determined according to PartitionFinder 2.1.1 [74]. MrBayes v3.2.6 [75] was used to construct the phylogenetic tree using Bayesian inference (BI) method based on the combined gene set. Two independent runs with four chains (three heated and one cold) each were conducted simultaneously for 2 × 10^6^ generations. Each run was sampled every 100 generations. We assumed that stationarity had been reached when estimated sample size (ESS) was greater than 100, and the potential scale reduction factor (PSRF) approached 1.0. The first 25% samples were discarded as burn-in, and the remaining trees were used to calculate Bayesian posterior probabilities (BPP) in a 50% majority-rule consensus tree [47]. We also construct the phylogenetic tree using maximum likelihood (ML) method based on the combined gene set using RA × ML v8.0.0 [76].

### 4.6. Availability of Data

The *R. salebrosus* and *R. vinicolor* mitogenome sequences were submitted to GenBank under accession number MH794152 and MH794153, respectively.

## Supplementary Materials

Supplementary materials can be found at https://www.mdpi.com/1422-0067/20/20/5167/s1.
