# Supplementary figures and images for: Comparative Mitochondrial Genome Analysis of Two Ectomycorrhizal Fungi (Rhizopogon) Reveals Dynamic Changes of Intron and Phylogenetic Relationships of the Subphylum Agaricomycotina
Source: Int J Mol Sci. 2019 Oct 18;20(20):5167. doi: 10.3390/ijms20205167 (PMC6829451; doi:10.3390/ijms20205167)

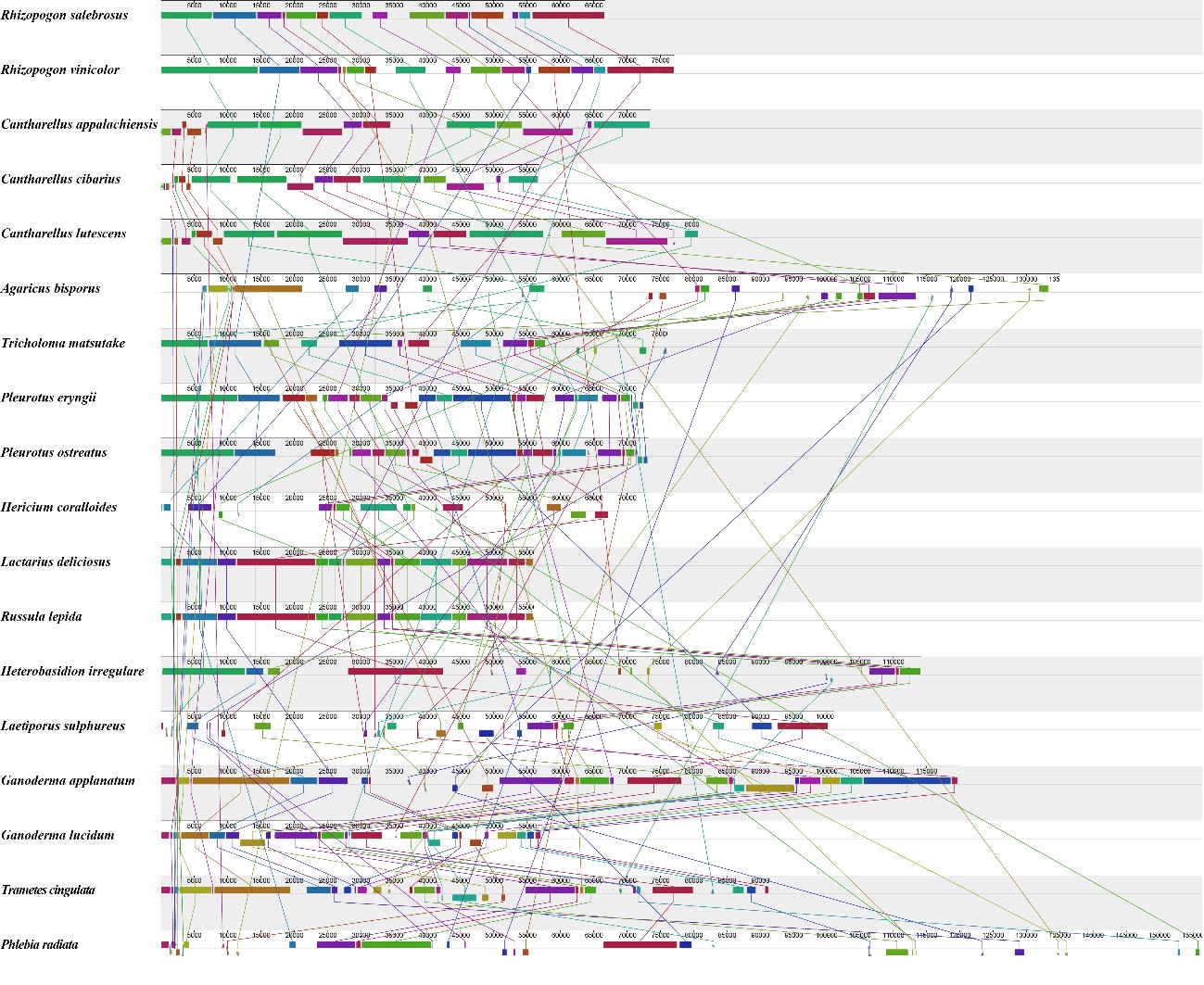

Supplement: Supplementary file 1 [file ijms-20-05167-s001.zip › ijms-610115-supplymentary/Figure S1. Collinearity analysis of 18 Agaricomycetes mitogenomes. Homologous regions between different mitogenomes are represented by same color blocks linked by lines..jpg]
